# The experiences of attempt survivor families and how they cope after a suicide attempt in Ghana: a qualitative study

**DOI:** 10.1186/s12888-017-1336-9

**Published:** 2017-05-11

**Authors:** Winifred Asare-Doku, Joseph Osafo, Charity S. Akotia

**Affiliations:** 1 Department of Psychology, University of Ghana, Post Office Box LG84, Legon, Accra Ghana; 2 School of Medicine and Public Health, University of Newcastle, Newcastle, Australia

**Keywords:** Experiences, Families, Cope, Suicide attempt, Ghana, Qualitative

## Abstract

**Background:**

Ghana’s socio-cultural landscape proscribes suicidal behaviour, and this has serious ramifications for attempt survivors and their families. The objective of this qualitative study was to understand the experiences of the families of attempt survivors and how they cope with the aftermath of the attempt.

**Methods:**

Ten families with attempted suicides agreed to participate in the study, hence they were interviewed. The information gathered was manually analyzed according to Interpretative Phenomenological Analysis (IPA) resulting in three major thematic areas.

**Results:**

Three major themes emerged two of which addressed negative experiences and reactions towards the attempts: *Experiencing shame and stigma,* and *Reactive affect.* The third theme addressed the coping resources of these informants under the theme: *Surviving the stress of attempt.* The specific coping resources included personalized spiritual coping, social support, and avoidance.

**Conclusions:**

Family systems theory is used to explain some of the findings of this study, and the implication for clinical practice and designing postvention programs for families after a suicide attempt are addressed.

**Electronic supplementary material:**

The online version of this article (doi:10.1186/s12888-017-1336-9) contains supplementary material, which is available to authorized users.

## Background

When suicide occurs, families experience a significant loss because they are those who are closest to the victim. Other experiences include pain, shame and distress with the potential for long-term effects including depression, suicidal ideation and other forms of distress have been reported [1]. For most families where a suicide attempt has occurred, shock is the first and immediate reaction [2]. Guilt feelings are also present in such situations especially when relatives regret things they did, said or did not do or say. Family survivors may feel that they directly caused the death, feel guilty, and blame themselves for not preventing the suicidal act [3]. In some situations, the family members express intense fear of another potential suicide in the family. Further, other affected family members may show physical symptoms like weight loss or gain, insomnia, pains and aches [4, 5]. For parents whose children attempt suicide, they may experience shock, panic and fear [4]. Shame has been shown to be one of the common experiences that characterize the aftermath of suicide attempt [6, 7]. Relatives experience shame from the stigma such behavior may bring onto the family. Stigma is a mark of shame and the stigma following suicide affects not only the suicidal individual involved but significant others and even future generations [1].

Usually in most suicide attempt cases, the medical condition is dealt with while the psychosocial factors are left unattended which include the experiences and how families and attempt survivors are coping after the attempt. Consequently, family members have to deal with the stigmatization and also provide care giving role for the suicide attempter who may be viewed as a cause of social injury to the family [8]. The suicide attempt may thus affect the relationship between families and the victim because of the social consequences of the act. The social living arrangement of Ghanaians is pervasively organized around interdependence [9]. The implication of this is that whatever affects one individual in the family affects the entire family [10].

Suicidal behaviour in general (i.e., thoughts, plans, attempt, death) are all condemned at three social institutional levels: family/community, religius and legal [11]. Reports of attempt survivors being either legally punished or socially ostracized have been reported in the country [12, 13]. Evidence continues to burgeon in support of decriminalizing or reforming the laws against attempted suicide in Ghana [14] and a call to empathize with attempt survivors and their families [15].

The distress of suicide attempt survivor families following the attempt, however, may vary from one cultural setting to the other. For instance, in Ghana and Uganda, suicidal behavior is considered a taboo and threat to the survival of the lineage, and thus has serious social consequences for the family [16, 17]. Accordingly, attempt survivors may be abused and stigmatized [12] . Such normative condemning attitudes and strict communal proscriptions may not be pervasive in other settings. For instance, in one study in the USA, attempt survivors reported excessive monitoring as reactions from families following their suicidality [18]. Such reaction might not be seen in the African (i.e Ghanaian) context. Reason being that a collateral damage is perceived, and thus the attempt survivor may rather be seen as a transgressor [11].

Several studies have examined the impact of suicide on the family in different countries [19–23]. In Ghana, there is dearth of such studies. A few studies have examined the experiences of suicide attempters only [12], but little is known about the experiences of the family following a suicide attempt, the nature of the reactions towards the attempt and how they cope with the attempt [24, 25]. This is the thrust of the present study. Families or family members as used in this article is exclusively related to any person in the family of the attempt survivor.

### Theoretical framework guiding the study

The Family systems theory by Bowen [26] is proposed as a theoretical framework to guide this study. Although based purely on western familial ideologies, the theory sits well with the African family structure patterned on communitarian arrangement [27]. The theory postulates that everything that happens to any family member has an impact on everyone else in the family. Individual human beings are therefore inextricably tied to their families [28]. This is because family members are interconnected, interdependent and operate as a group or as a family system. This connectedness makes the functioning of family members interdependent; hence a change in one person’s functioning will inevitably change the functioning of other family members. In some cultures, emphasis is placed on either individual or on the group [29]. Individualistic cultures are characterized by self-reliance whereas collectivistic cultures are more likely to define themselves in terms of being a member of an in-group [30]. Therefore individualistic cultures are more likely to see themselves as autonomous and collectivistic cultures see themselves as interconnected [31].

The theory further asserts that individual behavior cannot be understood in isolation, but should be examined as part of a larger system of which they are a part. Building on this, a family member’s suicidal behavior might have an impact on the functioning of other family members. The theory’s relevance might be crystallized in the Ghanaian context where social arrangement is patterned after communality [9]. In such a context, socialization patterns are aimed at censuring behaviors that bring losses to the collective family and rather encourage normative social behavior [27, 32].

## Method

### Setting

The research setting was located in Accra, within the Greater Accra Region of Ghana. Participants were recruited from the Psychiatry Department of Korle-Bu Teaching Hospital (KBTH) where all cases of suicide attempt presented to the emergency unit of the hospital are referred for psychological/psychiatric management.

### Participants

Participants were sampled using purposive and convenience sampling techniques, and also informants who will better be able to assist with answering the research questions and are readily available to participate in the study Table 1. The mean age for this study was 41 years and of the participants, 4 were fathers, 2 mothers, a brother, a husband, an aunt and a sister of the victims. The occupation of all the participants varied except two participants who were teachers. The others were a civil servant, trader, technical officer, security officer, dispatch rider, accounts officer and, carpenter.

**Table 1:** Demographic characteristics of suicide attempters and their families

Attempters survivors	*N* (%)	Family of attempters	*N* (%)
Males	4 (40)	Male	6 (60)
Females	6 (60)	Females	4 (40)
18–25	6 (60)	25–45	5 (50)
26–34	4 (40)	46–62	5 (50)
Student	5 (50)	Civil servant	5 (50)
Courier service	1 (10)	Artisans	2 (20)
Artisans	3 (20)	Security officer	2 (20)
Businessman	1 (10)	Courier service	1(10)
Poisonous substances	8 (80)		
Rope	1 (10)		
Slit wrist	1 (10)		
First timers	2 (20)		
More than once	8 (80)		

### Material and procedure

A semi-structured interview guide was developed based on contextual issues that emerged from previous suicide studies [8, 33, 34]. Some of the items on the interview guide included “*Have you had a suicidal attempt/death in your family”*, “*what do you think was the reason for the attempt”*, “*how did you react when you heard your relatives suicide attempt “tell me how you managed the feelings”?* Interviews took place at the residence of the participants and the premises of the Psychiatry Department. The interview sessions lasted between 40 min to an hour with the exception of one, which lasted for 17 min for lack of time on the part of the informant (family member). Four family members declined and refused to participate in the research. Of those who agreed, there were six males and four females, a total of ten (*N* = 10). Suicide is stigmatized in Ghana; therefore, the names of participants are not reported. Interviews were audio-recorded and transcribed verbatim (Additional file 1).

### Analysis

The Interpretative Phenomenological Analysis (IPA) was used to analyze the transcribed data. The main thrust of IPA is the meanings particular experiences, events, states represent for participants as means of elucidating how they make a sense of their personal and social world [33]. The focus was therefore on describing what all participants have in common as they experience the suicide behavior of some family members. The purpose of the analysis is to reduce individual experiences with a phenomenon to a description of the universal essence [35] and to explore in detail the lived experience of participants and the meanings they make of that experience. Firstly, the transcripts were read several times to become familiar with the data. Audio recordings were sometimes replayed along the reading of transcripts as well as some field notes on individual interviews. This active engagement was iterative and our purpose was to get into the lifeworld of each individual participant. Secondly, we noted significant statements, sentences or quotes that provided an understanding of how the participants experienced the phenomenon and also interpreted it. Thirdly, we reduced the provisional comprehensive explorative comments and notes into initial or emergent themes. Connections, interrelationships and patterns between these initial themes were sought at the individual level. Fourthly, we searched for how themes fit together across participants and produced a structure which allowed us to examine all the significant dimensions of the participants’ narratives and created a superordinate theme. We were mindful of context and how cultural beliefs and values might inform both the participants’ descriptions and our interpretations. Finally, we verified and summarized the themes, and established analytic connections across them. The researchers, fulfilled the double-hermeneutics underlying IPA analysis [36], by also interpreting the interpretations offered by the participants. Since the truth claims from an IPA analysis are often tentative and subjective [36], during analysis and writing we attempted to reduce bias and increase validity by scrutinizing themes thoroughly through discussions. Analysis was done by second author. The first and third authors critically challenged themes. Every theme was arrived at after a consensus. Consistent with Creswell and Miler [37] recommends that such cross-validation and group interpretations are useful tools to facilitate intersubjective comprehension, analytic rigor and trustworthiness of findings.

## Results

Three major themes were identified: *Experiencing shame and stigma*, *Reactive affect*, and *Surviving the stress of attempt*. For purposes of anonymity, we only report gender and relations of the participant to the attempt survivor (e.g., M-male and F-female, Father, Mother,etc) and age with the quotes.

### Experiencing shame and stigma

Shame was a common reaction some family members experienced following the suicide attempt of a relative. In one case, shame discouraged a family member from seeking help from anyone including the extended family for fear of stigma. This shows the layers of stigma: intra-family stigma, extra-family stigma. The pain experienced from the attempter’s action was not allowed to go beyond the confines of the nuclear family. The narrative below is illustrative.

*Actually I was ashamed, when it happened. That’s why I find it very difficult … to tell you. I have never ever told any of my family people, I have never. So it’s just between me and the nuclear family. I have never told any of my sisters and brothers or whatever. I have never spoken to them that he (the attempt survivor) is even in the hospital or something. So it’s just me and my wife who know it but my wife brought her sisters around. I didn’t understand (Father, 59 years).*

The above narrative further illustrates the gender dynamics involved in the management of the shame following suicide behavior in the family. The informant, who is the father of the attempter, was making frantic efforts to contain the incident “indoor”, but the wife (mother of the attempt survivor) sought support from her siblings. This attitude was corroborated by another male informant of keeping the incident silent: *This is not something you tell people about, it’s a bad thing that happened and I know it just something that is passing. Even my sisters I didn’t tell them (Male, 58 years).* The differences in reactions to the incident of suicide in the family by gender might be consistent with the general knowledge that women seek help promptly compared to men [38, 39].

Other informants were fatalistic in the way they experienced the shame and were unenthused to do anything to salvage the situation as illustrated by the following quote: *“I felt sad and ashamed and the harm has been done already so there is nothing to do about it... Very pathetic, very pathetic. It’s very sad…very very” (Mother, 56 years).* The “harm” the informant refers to, does not appear to be a physical one, but rather psychosocial; the harm of shame to the family. She presents this harm in superlatives to emphasize perhaps the degree of damage the act might have inflicted unto the family. The potential damage inflicted on the family is presented as irreparable and may be a fruitless endeavor to seek redress. The attempt survivor’s life is not a core issue, compared to the family’s image.

For some family members, their emotional pains after the attempt emanated from stigma from their community. As explained, the kind of stigma community members expressed derives from the enigma surrounding suicide as indicated below:

*That’s why I said earlier that I sometimes feel rejected by people because they didn’t understand why such a thing should happen in our family. People are not happy at all, some withdrew from us, and the stigma associated is not easy. We are still in a small community and such rural communities the stigma is great unlike urban areas.*

The physical expression of stigma is seen in avoiding interacting with the surviving family. This he explains as reflecting the nature of stigma from rural communities. The closely knitted living and social arrangements might make any attempt to hide a suicide incident from others almost impossible. Other further important issues are expressed in the narration of the informant:

*The signs of the attempt are still there so we are still living with the stigma. The sign, I mean the scar on the neck. It is visible and everywhere she goes people see the scar and it reminds them even when they have forgotten. You see that when she came to see you she (the attempt survivor) had a scarf; yes the scar is very visible. Some people in the community even try to provoke her and ask her about it. The act is an abomination in our community (Husband, 44 years).*

It appears, as can be deduced in the above quote, that when one cannot be de-individuated within a small nexus of social group, the stigma of suicide in a rural setting bites deep. This is illustrated by the direct link he establishes between the physical scar from the wound sustained, and the memory of the community members. He conceives the scar as an indelible mark of stigma, which continues to remind the community members of a suicide attempt in the family. His reasoning appears to establish a consistent connection between the tabooed nature of suicidal behavior and the consequential communal stigma.

### Reactive affect

This theme addresses the emotions informants expressed towards the incidence of suicide attempt from their relatives. Some informants expressed shock, surprise, and anger at their relatives who attempted suicide. The expression of surprise for example was apparent in the face of shattered myth. For instance, some held the fable that lack of needs might be a precipitant to suicide and thus when the needs of someone are fully met there should not be suicidality. This is gleaned from the following quote: *I give her everything she wants and am surprised myself that something like this will happen (Father, 62 years)*. The expression of surprise is therefore a product of ignorance and reflects a knowledge gap of the nature of suicidality. In some instances, a participant expressed shock but not surprise. Shock and surprise might be forms of emotional arousal so close to delineate, to the extent that to admit the occurrence of one without the other might be contradictory. But in one case a husband expressed shock, simultaneously saying he was not surprised when the wife attempted suicide:

*Actually I was shocked when it happened. I was not surprised, because I saw the signs and saw it coming. I even sometimes talked about it with some few people. People thought I was only trying to demonize or make my wife appear bad. I was shocked and till date I haven’t fully recovered from the shock (Husband, 44 years).*

This seeming contradiction could be addressed by taking a closer look at the medical history of the attempt survivor as narrated by the participant. The attempt survivor (wife of this informant) has been diagnosed with borderline personality disorder. It is plausible that he (the husband and participant) was living with the expectation that a suicide attempt was inevitable. This might explain his lack of surprise. However, his shock could result from the reality of the near death of a spouse. Self-preservation instinct makes self-destruction difficult but the ability to subvert this could be shocking to those who are unaware of the nature of suicidal behavior. This contradiction, thus, on one hand reflects some level of ignorance on the relationship between mental illness and distress, and suicidal behavior.

In another case, the shock and surprise appear to be driven by a perception that the act constituted a waste of resources and potential. The attempt survivor had finished her secondary examinations, the passing of which may grant her access to pursue a university education. In Ghana, a low and middle-income country, achieving such a feat is a milestone, an honor to the family, and thus worth celebrating. Yet from the father’s perspective, such a feat appears denigrated by the daughter’s self-destructive tendency:

*I was surprised and shocked because she had just finished writing her WASSCE (West African Senior Secondary Certificate Examination) a week ago when this happened. I was thinking that maybe someone has realized that she is going to be a great person in future and wants to eliminate her. I do not have any bad intentions towards my daughter. When their mother died, she was 8 years old and I have taken care of her and her brother till now. I was sad because I have taken care of her from when she was born until now and she wants to just kill herself (Father, 46 years).*

The shock perhaps would have been manageable if the father’s suspicion of diabolical interference was a credible explanatory alternative. Yet he seems to have given up on that conspiracy to accepting sheer agency in the attempt; the daughter’s own will. He finds this surprising and perhaps compares the fortitude he had mustered to endure painful moments such as loss of his wife to the perceived reckless act of his daughter (the attempt survivor). He may consider his resolve to thrive, a great sacrifice, and a virtue undeserving of such action (suicide attempt) from the daughter. Some perceived sense of ingratitude (from the attempt survivor) can be deduced from the latter section of the father’s narrative.

Anger was another emotion that was often expressed by the informants toward a relative’s suicidal act. In some cases, the anger towards the attempt survivor was a vendetta to the perceived social wound inflicted on the family, a situation that they are arduously adjusting to:

*I sometimes feel angry because the incident has put us in a very difficult situation in the vicinity where we live and we have still not recovered much yet. It has changed our family course and I know that it will take us several years to recover very little (Husband, 44 years).*

As indicated above, the impact of the suicide attempt is perceived to have inflicted a significant damage on the wellbeing of the family and that recuperation will take lot of time. He continues the narration:

*...But that is not the reason why I am angry, but the fact that the recurrent things are still there. But I know the anger is only an imperfection within me because I know in the normal frame of mind she (the attempt survivor) will not do it. I know that perfectly. But sometimes looking at the situation especially when her emotions come and she is not behaving well, I get angry but I control it. (Husband, 44 years).*

The fundamental motive driving the anger as indicated above is the lingering of potential signs of repeated attempts. Clearly, such thinking might hamper family support for the attempt survivor. This fixation with repeated attempt may create anxiety in the informant and hamper the development of a supportive relationship for the attempter.

### Surviving the stress of attempt

This theme addresses how attempt survivors family members coped with the stress from the suicide attempt. Various coping mechanisms emerged: personalized spiritual coping, social support, and avoidance.

Some of the informants indicated using spiritual means to cope with the distress following a member’s suicide attempt. For some, such spirituality is mixed with withdrawal from any human support. The withdrawal is explained as reflective of commitment to religious injunction as illustrated by the voice below:

*I cope by not telling people. When people hear of it, they will be gossiping and in future they will be insulting you. I pray too, as for prayer, prayer is the key. I tell my God, I don’t tell human being. That is what we have been taught to do. They said we should talk to our God (Mother, 56 years).*

The informant mixes prayer with some level of paranoia. Her background showed that she had lost a cousin through suicide and was socially stigmatized. Now her son in medical school also attempted suicide. These experiences appeared to have influenced her adoption of an unpopular method of coping with life’s stressors: silence and masking. Such coping method as can be gleaned from her narrative was to avoid any potential verbal abuses. What is further interesting is how she links this experience to scriptural injunction. In her lay theology, true prayer is one that is directed toward God without regard to seeking any human intervention or help seeking. Within the ambit of such theological understanding, her silence and masking coping strategy finds religious validation.

In another case, the informant used prayer as coping strategy. His dependence on prayer was intensified when a sibling also collapsed few days after the senior one attempted suicide:

*As for prayer, I really prayed because she (attempt survivor) had just finished Senior High School (SHS) when she attempted suicide. My other child is at Kwahu SHS and also writing WASSCE (West African Senior Secondary Certificate Examinations). He was home last week, and I don’t know what happened, and he just collapsed. This made me pray more because the devil is a liar. He was discharged the following day (Father, 46 years).*

The informant suspects diabolical interference in the attempt. This was accentuated by the unexplained collapse of the other child. The validity and efficacy of his prayer is found in the belief that his prayer might have hampered diabolism and ultimately facilitated the discharge of the son from the hospital.

In other forms of spiritual coping, an informant mixed prayer, prosocial acts, and religious involvement as can be seen from the quote below:

*Praying and visiting people made me feel better. The major one is my involvement in religious activities. Our family identifies with the Jehovah Witness religious group and the teachings on love, morality and others have helped me. I learned that difficulties are present in life and we must learn how to manage them. (Husband, 44 years).*

The informant in the above quote indicates that religious teachings on love and morality have shaped his understanding of managing life’s struggles through prayer and visiting people, but the greatest of all is immersing himself in religious activities. He has achieved a state of meaning: that life is inherently driven by struggles but the essence of survival is rooted in managing these struggles. His prosocial tendency is consistent with other studies that seem to support the notion that exposure to stress increases prosocial tendencies such as trust, trustworthiness and sharing behavior in social interactions [40]. Further, evidence has established a positive association between the frequency of church attendance with well-being and a negative one with distress [41].

Other informants confided in friends and family and this helped them to cope with the stress associated with the attempt. Whereas one informant acknowledged that he did not have any close friend who supported him in the aftermath of the suicide attempt, his friend sympathized and inspired hope in him*: The only friend I have is just casual friends, “boys boys”. But don’t really have a tight friend. I told them about it and they encouraged me that it will be well (Male, 46 years).* An informant indicated that he managed the stress well because his friend engaged him. From his narrative, the friend used humor to soften the seriousness of the situation. Further frequent visits from his friend coupled with a sense of hope helped manage his distress as he shared below:

*Am having a best friend, he was visiting me and making funny comments so I laugh and forget about the situation. He comes to me always. (Brother, 25 years).*

Social supports did not only come from friends but also from families. Some extended and nuclear family members were informed about the suicide attempt and they responded to provide support for the affected members. Their response could be viewed as an obligation to reciprocate the privilege granted them of hearing the news of a suicide attempt: *Family members were there to help me cope. I didn’t tell anybody apart from family. It was only my family that was around (Father, 62 years).* For others, the support came from close family members such as children and siblings. Perhaps this is a description of close family, the nuclear family that should hear some news and may be expected to provide help as indicated below:

*Only my two daughters and mum were helping me to cope, they call every day and assist me in many ways. My other siblings didn’t call me or check up on us after the incident. I don’t have any friends because am always busy with work and my mother (Mother, 56 years).*

It appears the participant was complaining that her siblings have abandoned the nuclear family. This response reflects the aftermath of the attempt as she intimated. What is evident in this is that the nuclear family is the source of most of the support she received than from any other extended social relations.

## Discussions

The present study was aimed at examining the experiences of family members of attempt survivors and how they cope with the stress associated with the attempt. First, as reported by some previous studies, attitudes toward suicide attempt continue to be negative and both the experiences and reactions of family folks towards suicide as reported in the present study are consistent with those studies [1, 8, 12, 34]. The analyses further showed that the participants were experiencing shame with feared stigma following the suicide attempt of their relatives. Suicide stigma is deep in Ghana and has been reported to be institutionalized communally, religiously and legally. Accordingly, the suicidal person is viewed as antisocial, transgressor or sinner, and criminal [11]. Suicide stigma in Ghana is believed to be injurious and contagious and persons related to the victim may avoid the victim’s company [8]. Plausibly, the indications in the present study, that some suicide survivor families were frantically trying to contain the news and prevent it from circulating might reflect this cultural aversion to suicide stigma. In Uganda, for instance, the entire family distances themselves from the suicidal person as an act of ritually managing the impact of a collective social damage following the act [42].

Attempt survivor families also reported bouts of negative emotions such shock and anger as reported in some previous studies [4, 7]. Emotions are processes of appraisal of events either positively or negatively and it is important to look at these emotions within the purview of dyadic aspects of suicide. Emotion is also related to social cohesion as they may separate individuals from others or may join them to others [43]. Therefore, these emotions experienced by the families may break social cohesion between attempt survivors and their families. From the findings of the study, negative emotions do affect the interpersonal relationship between the family and the attempt survivor, a relationship that is mostly needed for the support of the attempter. In one study in Ghana, an attempt survivor eventually killed himself following increased social taunting and verbal abuse from the rest of the community and family folks [12]. If a history of attempt elevates the risk 40 to over 100 times compared with that in the general population [44, 45], then there is the need for special relationship and communication with attempt survivors with their close relations of which family members are key. A cultural context such as Ghana, rife with condemnation against suicidal behaviours may hamper any of such prosocial tendencies of attempt survivor families when they are needed most. While some studies postulate that family folks are prevented from offering helpful reactions following suicidal behaviour due to lack of understanding of suicide and being overwhelmed by emotions [46, 47], the present study adds one more complexity to this: the dynamic role of cultural conceptions about suicide and suicide stigma. The cultural contexts of suicidality are critical issues in current suicidological discourses and their role in understanding suicidality and its prevention cannot be underrated [48, 49].

Some important issues in defining and conceptualizing suicidal behavior have been asserted [50]. Shneidman indicates that one important conceptualization of suicide is that most suicidal acts are dyadic events with two phases [51]. In phase one, one has to deal with the “significant other” and this is during the prevention of suicide. In phase two, one has to deal with the aftermath of a suicide. Here one must deal with the survivor-victim relationship. The present study, although not dealing with suicide and attempt survivors (but families of attempt survivors), the nature of the negative reactions of the family and relationships with the attempt survivors following the act can be postulated within the survivor-victim dyad; or perhaps the attempt survivor-family dyad. The suicide attempt, though an individual action, may involve and affect the entire family dyad. These may include, parent and child, sibling and sibling, spouse and spouse, lover and lover. This study has involved some of such dyads: parent and child, lover and lover. Such dyads are extremely important to manage since they might provide the platform for the escalation of perturbation. The aftermath negative reactions such as shame, shock, guilt and anger as discovered in this study portend further dyadic crisis. As long as the victim may be conceived in moral terms as an offender, such dyadic tensions can continue to fuel perturbation leading to eventual suicide. This is an important point since the single most important risk factor for suicide is attempted suicide [52] and thus family members reactions following the act should be carefully managed and guided to rather improve attempt survivors’ recovery.

The study further showed that attempt survivor families did not receive much help following the attempt. This might reflect both self-stigma (which in some of the cases discouraged informants from seeking help), lack of credible institutionalized professional support and the generalized negative attitudes toward suicide in Ghana. Perhaps such widespread stigma forced the family survivors to avoid seeking help from congregational support in their respective churches. They rather turned to individualized spiritual coping mechanism. Religion is one of the influential social forces in Ghana, which institutionalizes the stigma against suicide [12]. The lack of confidence some informants expressed in seeking help from organized religious groups is an indication that the fear of stigma is leading to isolation and impairing the social network of these family survivors following the act [53]. This stigma may add to the trauma and may make healing of the family survivors’ and even the attempt survivors slow and arduous with potentially poor mental health outcomes. There were other suicide attempt family survivors who received social supports from friends and family folks and is consistent with the important role identified by families and friends in the treatment following suicide attempts [54].

These findings could be further explained within the postulations of the family systems theory [26]. As individuals are inextricably linked to their families, a network of interconnectedness is established. Thus from a systems perspective, a change in an individual’s functioning affects everyone in the family. In the present study, the suicidal behavior by a family member negatively affected the lives of other family members. This is consistent with the social injury conception of the impact of suicidal behavior in Ghanaian families [8]. Thus in the context of this study, suicide is a familial and social issue in Ghana more than a personal and individualized issue.

### Implications for clinical practice, research and training in Ghana

There are important implications of the findings in this study for clinical practice and research in Ghana. From the findings of this study, individualized psychotherapy for only attempt survivors leaving out their families might not be useful. The dysfunctional social relations that may develop between attempt survivors and their families are critical indicators for initiating family therapy sessions for attempt survivors and suicide literacy for their families in Ghana. Joiner’s interpersonal theory of suicide contends that failed belonginess and perceived burdensomeness are risks for suicide [55]. Families may be needed therefore to provide ‘suicide watch’ in crisis situation, but this very important assignment could be hampered by negative emotions experienced, following the act [54]. Treatments of attempt survivors should emphasize how dysfunctional family relationships could further affects both family member and the attempt survivor. Therapy sessions involving families could accordingly, help clarify and ease tensions thereby reducing further risks. Families should therefore receive educations on how to be communicate with the attempt survivor as well as how to manage their own stress of being aware of the suicidal crisis of their relative [54]. Thus a postvention program should provide the family with educational resources to equip them, assess their feelings, reactions, and coping methods so that appropriate referrals and services can be provided. The family context is a major suicide prevention resource and such education can target reducing myths, poor attitudes, and stigma and improve the early identification of warning signs [50]. The indication that some family members (informants) were caught off guard, held unto some fables about suicide and attributing diabolism are key knowledge gaps that should be addressed in light of this recommendation through sound research that explores stigma, suicide illiteracy and how it impedes early intervention in research at the micro level (such as family).

In terms of training, affected families and non-affected families, as well as religious groups and (and their leaders) can be targets for gatekeeper training and suicide literacy. Adopting the Train-the-Trainers (TT) approach [56] can help provide education on suicide prevention for these groups who can help persons in suicidal crisis receive early help. This is consistent with the important need for gatekeeper training, as prevention and intervention technique, identified by WHO [52] report on global suicide prevention.

## Conclusions and limitations

In conclusion, this study found that firstly, social relations in Ghana bereave and feel morally offended by the suicidal act of a relative since it constitutes for them a social injury [8]. This injury is further deepened due to the stigma attached to suicide in Ghana as was evident in the narrations by families.

Secondly, this conception provokes negative emotions such as anger and shock which further foment dyadic tensions between survivor and victim leaving each emotionally distressed and unattended. Thirdly, there are no support systems for family survivors as stigma (both self and societal) discourages them from seeking help from within their communities (e.g., religious).

This study was exploratory in nature, providing a sort of baseline for further research into the familial context of suicidal behavior in Ghana. Families do experience emotional distress, which may degenerate and create further distress for the attempters with risks for suicide completion and yet coping behaviors of families are largely influenced by cultural dynamics. Suicidal behavior continues to be a cultural artifact and any prevention attempts should seriously consider cultural issues which permeate attitudes, reactions, and coping in order to provide culturally sensitive programs on suicide prevention [48].

A major limitation of the study was that, due to the sensitive nature of the topic some families were not interested in participating. It is a seminal study seeking to document relevant experiences of families of attempt survivors to guide intervention. The lack of a good number of families unrepresented does deny the study potentially relevant information in this area. Further, the study was time-bound and therefore we could not spend all the time on the field seeking voluntary participation. Future studies can consider a longitudinal approach to uncover more cultural dynamics in the experience of suicidality from the perspectives of families and how this can impact on suicide prevention in the country.

## Additional file

Additional file 1: Interview guide. (DOCX 13 kb)
