# Supplementary material for: The experiences of attempt survivor families and how they cope after a suicide attempt in Ghana: a qualitative study
Source: BMC Psychiatry. 2017 May 11;17:178. doi: 10.1186/s12888-017-1336-9 (PMC5426021; doi:10.1186/s12888-017-1336-9)
Supplement: Additional file 1: — Interview guide. (DOCX 13 kb) [file 12888_2017_1336_MOESM1_ESM.docx]

- - - 1. File name: Interview guide

*1) The interviewer briefly presents the background for the study and the structure of the following interview.*

*2) The terms of the informant’s voluntary participation is repeated as well as the possibility to withdraw without consequences at any time. Confidentiality and anonymity is also assured.*

*3) The tape recorder is introduced and demonstrated.*

*4) Declaration of informed consent is signed (or oral consent obtained in case of illiteracy).*

*The tape recorder is switched on.*

**Sex**

**Age**

**Profession**

**Years in profession**

**Tribe/ethnicity**

**Religion**

**Marital status**

**Presently living with**

**Area of residence**

**Relationship to suicidal person (for survivors)**

**When did incident occur**

1. **General questions**
   1. How prevalent do you think suicidal behaviour is in Ghana today?
   2. Do you think it will change in the future?
   3. If so, in what direction and why?
   4. How is the topic of suicide handled/viewed in Ghana today?
   5. In general (do people talk about it, how is the general opinion, etc.)
2. **Specific questions about suicide experience**
   1. Have you had a suicidal attempt/death in your family? (probe when, how , who etc)
   2. What do you think caused the reason for the attempt?
   3. Which warning signs did you observe?
   4. Tell me the threats the person issue?
   5. Tell me if there were any life stressors the person was experiencing prior to the attempt
   6. Tell me if attempts were made to let the person see a helper (probe: who was the helper? When was the helper seen? What did the helper recommend?)
   7. Tell me the help you (or any family member) provided for the person at this stage
3. **Reactions and coping**
   1. Tell me how it feels to hear a close relation has attempted suicide
   2. Can you please describe how people reacted to the attempt?
   3. Can you please tell me how your family folks reacted to the attempt?
   4. Tell me how you managed these feelings (refer to the feelings named previously) after the attempt?
   5. Were there people helping you to cope and who were these persons (probe: how did they help you?)
   6. Please tell me the forms of support available from your church? (Probe: did you observe any stigma reaction from the church?).
   7. Can you please explain to me the difference between the support you received from others (e.g., friends, family, clubs, organizations you belong to) and your church?
4. **Closure and debriefing**
   1. Do you have anything else to tell me about this topic?
   2. Do you have any questions for me?
   3. How did you feel about being interviewed on this topic?

Is there anything you wish had been done differently?
